# Sensors for Fire and Smoke Monitoring

**DOI:** 10.3390/s21165402

**Published:** 2021-08-10

**Authors:** Robert S. Allison, Joshua M. Johnston, Martin J. Wooster

**Affiliations:** 1 Centre for Vision Research, Department of Electrical Engineering and Computer Science, York University, 4700 Keele St., Toronto, ON M3J 1P3, Canada; 2 Canadian Forest Service, Great Lakes Forestry Centre, 1219 Queen St. E., Sault Ste. Marie, ON P6A 2E5, Canada; Joshua.Johnston@canada.ca; 3 Leverhulme Centre for Wildfires, Environment and Society, Department of Geography, King’s College London, Aldwych, London WC2B 4BG, UK; martin.wooster@kcl.ac.uk

Mastery of fire is intimately linked to advances in human civilization, culture and technology. It is also a key component of renewal and regeneration of natural ecosystems. At the same time fire continues to be a threat to lives, property and the environment. Our complex relationship with fire has been the impetus for many innovations in direct and remote sensing of key properties of fires including flame, heat and smoke. This Special Issue on fire and smoke detection and monitoring has curated and collected the latest research on sensors and systems to detect and quantify wildland, structural, and industrial combustion, and the emissions of smoke produced.

Public interest surrounds detecting and monitoring fire and smoke behavior for fire protection and suppression in residential and industrial settings, or for the design and test of safer materials and structures. The time lag required to detect smoke in a building fire is a key factor in the likelihood of harm to occupants. Jang and Hwang [1] carefully controlled smoke velocity and concentration to measure obscuration thresholds for different detectors with different fuels. They found that sensitivity of photo-electric and ionization sensors depended on the type of fuel. The results will be useful to predict detector activation times and determine the required safe egress time.

The majority of the papers concentrated on wildland fires. This is likely due to a combination of a large number of concerted research programs and recent advances, combined with increased public interest. The devastation from the recent wildfires in California, Australia, and elsewhere has captured the attention of the public, government officials and funding agencies and highlighted the need to detect and monitor wildfires to protect and preserve life and property. Image-based remote sensing from the air and space across different wavelength regions has revolutionized the monitoring and management of wildfires (for review see [2]). Barmpoutis and colleagues [3] review the current state-of-the-art in optical remote fire detection systems from terrestrial, airborne or space platforms and present a bibliometric analysis of the literature in addition to their review.

Recent advances in low resource detector technology and the rise in the availability of data from small satellites has seen a surge in innovative detector technologies and in the applications to which such data are put. Dufour and colleagues [4] present an innovative sensor system for measurements of biomass burning. The system is based on a bi-spectral microbolometer that can make radiometric measurements at long- and mid-wavelength IR. Such a system would enable low-resource sensor platforms such as nanosats or small UAVs for wildfire monitoring. Johnston et al. [5] detail the system features and performance requirements for a wildfire monitoring satellite in the context of the WildFireSat mission. The user-centric systems approach illustrates the complex interplay of observational, measurement and precision requirements for a successful and useful system.

Progress depends on the ability to extract meaningful information from the sensor systems, and many papers focused on processing of the sensor data to extract meaningful fire characteristics. At the experimental level, Dickson et al. [6] provided new insights into fire dynamics through careful analysis of direct measurements to better characterize sensible heat flux within a spreading flame-front. Fisher et al. [7] were able to conduct regional analysis of particulate emissions from El Niño exacerbated wildfires in Indonesia using geostationary satellite observations, expanding our understanding of the contribution of different types of fires to these types of harmful emission during severe fire events.

Processing of sensor data and classification of such data with limited resources was considered by several papers. Ifimov and colleagues [8] were confronted with a mid-wave IR airborne image dataset without reliable geolocation data. They were able to use a two-step semi-automatic geocorrection process to generate informational products such as fire radiative power density (FRPD). This technique could be used by other researchers to process airborne infrared imaging data without the need for highly accurate positioning data. Sousa et al. [9] focus on mobile platforms and the integration of off-the-shelf thermal imaging cameras into mobile robot image processing pipelines. Tlig et al. [10] adapt PCA-based image processing techniques to extract relevant fire management characteristics from wildfire imagery. Pan et al. [11] apply convolutional neural networks for forest fire detection and prune the network based on Fourier domain similarity to reduce the resource requirements for edge computing implementations of the technique.

This Special Issue presents a unique cross section of current research priorities related to fire and smoke sensing in the scientific community. Although far from a complete survey, the articles presented demonstrate a growing interest in the use of remote sensing for wildfire research and monitoring, and the pursuit of new technologies to broaden the potential of this field. Notably, there is a strong interest in processing techniques for extracting increasingly complex products from existing data types. However, the use of direct measurement sensors and the careful analysis of the data they produce continues to provide new insights into wildfire dynamics and human safety in domestic settings. It is the view of the guest editors that this is an accurate reflection of the present focus of our field, and that this Special Issue is a suitable introduction to the topic of Sensors for Fire and Smoke Monitoring. 
